# Targeting the Sarcomere: Myosin Inhibitors as the Revolutionary Game Changer in Hypertrophic Cardiomyopathy

**DOI:** 10.31083/RCM47341

**Published:** 2026-01-22

**Authors:** Farbod Sedaghat-Hamedani, Elham Kayvanpour, Benjamin Meder

**Affiliations:** ^1^Department of Cardiology, Angiology and Pneumology, Institut für Cardiomyopathien Heidelberg, University of Heidelberg, 69120 Heidelberg, Germany; ^2^German Center for Cardiovascular Research (DZHK) Partnerside Heidelberg, 69120 Heidelberg, Germany; ^3^Precision Digital Health and Informatics for Life, Clinic of Cardiology, Angiology and Pulmonology, University of Heidelberg, 69120 Heidelberg, Germany

**Keywords:** hypertrophic cardiomyopathy, myosin inhibitors, myosin heavy chains, mavacamten, aficamten, precision medicine

## Abstract

Hypertrophic cardiomyopathy (HCM) represents the most common inherited cardiac disease and a leading cause of heart failure, arrhythmias, and sudden cardiac death in young individuals. For decades, management of HCM has relied on symptom control with β-blockers, calcium channel blockers, disopyramide, or invasive septal reduction in advanced cases. The identification of pathogenic sarcomere variants and the recognition of hypercontractility as a central disease mechanism have paved the way for cardiac myosin inhibitors (CMIs), the first truly disease-specific pharmacological therapy for HCM. Indeed, CMIs represent a revolutionary therapeutic paradigm that redefines the standard of care by translating molecular discovery into clinical application. This review provides a guide to the mechanistic basis of sarcomere modulation, summarizes the clinical evidence for mavacamten and aficamten, and critically evaluates the evolving roles of both medications in obstructive and non-obstructive HCM.

## 1. Introduction

Hypertrophic cardiomyopathy (HCM) is the most prevalent inherited cardiovascular disease, affecting approximately 1 in 200–500 individuals worldwide and representing a leading cause of heart failure, arrhythmias, and sudden cardiac death in the young [1, 2]. Despite significant advances in genetic mechanisms leading to HCM, treatment strategies have for decades remained confined to symptom control using β-blockers, non-dihydropyridine calcium channel blockers, and disopyramide, or invasive septal reduction therapy in refractory cases [3, 4]. These approaches, while effective in reducing symptoms and outflow gradients, fail to address the fundamental molecular mechanism of HCM: sarcomeric hypercontractility, increased binding probability of actin-myosin and inefficient energetics.

**Fig. 1.**
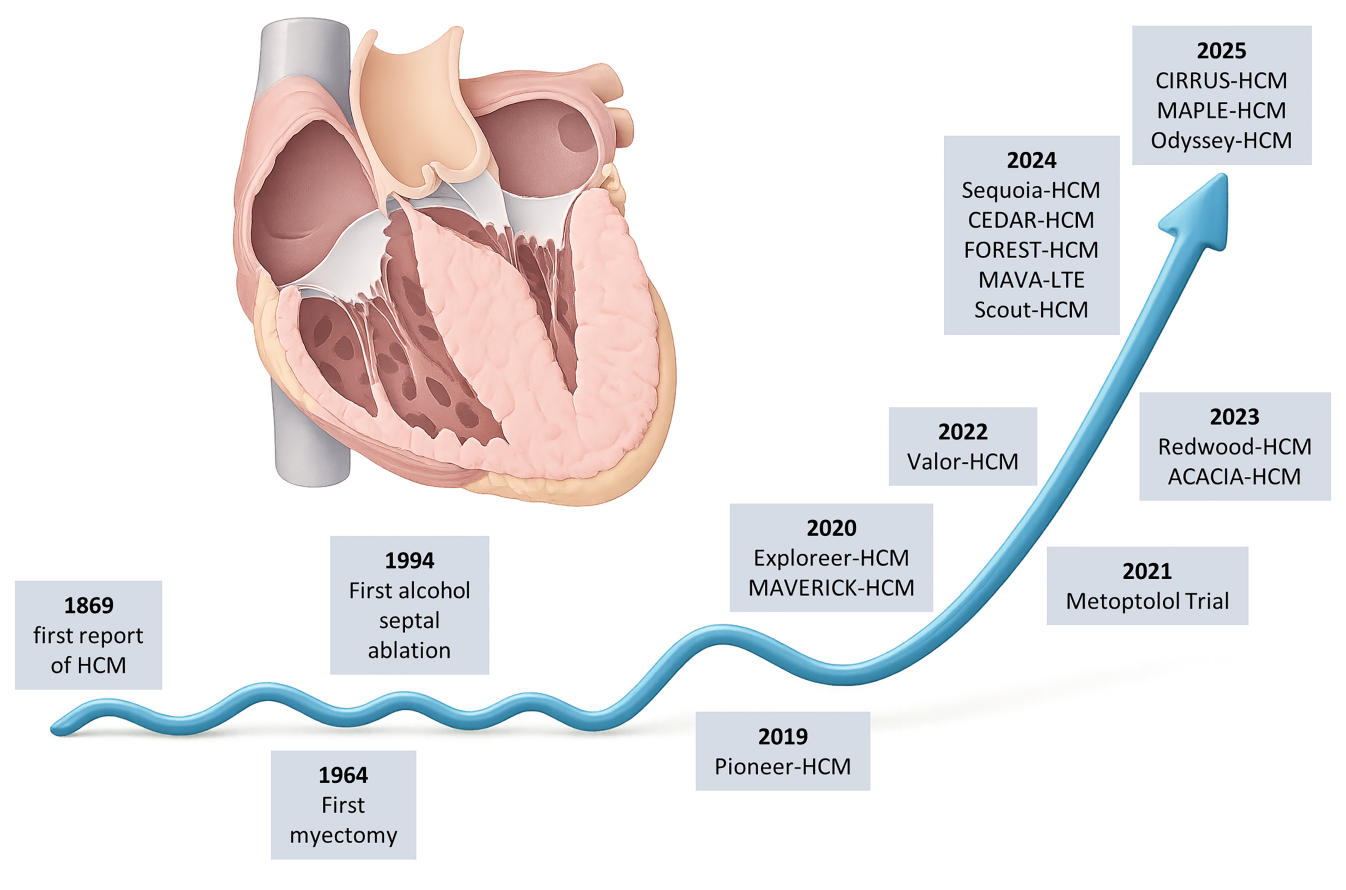
**Timeline of milestones in hypertrophic cardiomyopathy (HCM)**. Key advances are shown from the first clinical description (1869) and early surgical/interventional therapies (myectomy, 1964; alcohol septal ablation, 1994) to the modern era of cardiac myosin inhibitors, with landmark trials illustrating the paradigm shift from invasive to disease-specific pharmacological therapy. Created in Illustrae (https://illustrae.co/)

The identification of pathogenic variants in sarcomere genes, particularly myosin heavy chain 7 (*MYH7*) and myosin-binding protein C (*MYBPC3*), established HCM as a primary disease of the contractile apparatus [5]. This recognition shifted therapeutic aspirations from symptomatic palliation toward disease-specific intervention. Over the past decade, insights into cross-bridge kinetics and actin–myosin interaction have culminated in the development of selective cardiac myosin inhibitors (CMIs). By directly modulating myosin ATPase activity, these agents reduce excessive cross-bridge formation, increase myosin in super-relaxed state (SRX) thereby normalize contractility, and mitigate left ventricular outflow tract (LVOT) obstruction [6]. Mavacamten, the first-in-class myosin inhibitor, provided proof-of-concept that targeted sarcomere modulation could alter the natural history of HCM. Parallel development of aficamten, a next-generation myosin inhibitor with different pharmacokinetics, has reinforced the therapeutic potential of sarcomere modulation [7]. Together, these advances mark a paradigm shift in the management of HCM. For the first time, a pharmacological therapy targets the underlying molecular pathophysiology rather than its downstream consequences. Myosin inhibition has thus emerged as a revolutionary game changer, bridging the gap between genetic discovery and clinical translation (Fig. 1). This review summarizes the historical evolution of myosin inhibitors, synthesizes evidence from pivotal trials, and highlights future directions including use in non-obstructive HCM, pediatric populations, and novel sarcomere-targeting agents.

## 2. Mechanistic Basis of Myosin Inhibition 

The defining hallmark of HCM is sarcomeric hypercontractility, a direct consequence of pathogenic variants in genes encoding thick and thin filament proteins, most notably *MYH7* and *MYBPC3* [8]. At the cellular level, these variants increase the proportion of myosin heads available for actin interaction, thereby augmenting force generation but impairing diastolic relaxation and myocardial energetics [6, 9]. For mutation elusive obstructive HCM, the mechanism is not as well understood, but response to myosin inhibition seems comparable as shown in recent clinical trials. The resulting hyperdynamic contractile state promotes left ventricular hypertrophy, microvascular ischemia, fibrosis, and ultimately heart failure [10, 11, 12].

Cardiac myosin exists in a continuum of conformational states. In the SRX, myosin heads are sequestered along the thick filament backbone, minimizing adenosine triphosphate (ATP) turnover and conserving energy. In contrast, the disordered relaxed state (DRX) exposes myosin heads, increasing their probability of actin engagement and cross-bridge formation [13, 14]. Genetic variants destabilize the SRX conformation, shifting the balance toward DRX, thereby driving hypercontractility and energetic inefficiency [14, 15].

**Fig. 2.**
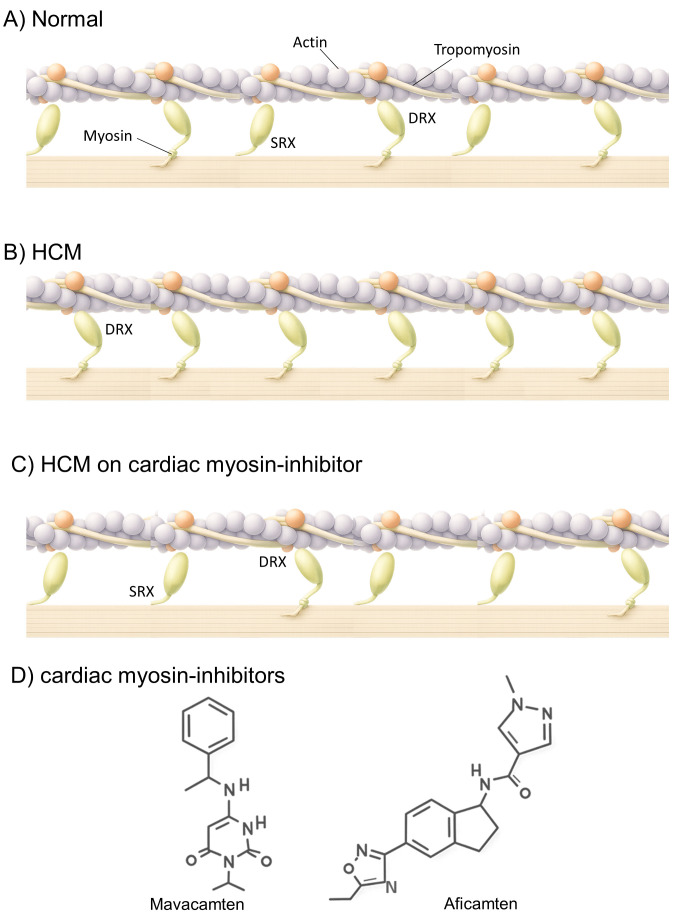
**Mechanism of action of cardiac myosin inhibitors (CMIs)**. Balanced distribution of myosin heads between the super-relaxed (SRX) and disordered relaxed (DRX) states, permitting normal contractility and efficient relaxation (A). In hypertrophic cardiomyopathy (HCM), destabilization of the SRX state increases myosin head availability for cross-bridge formation, resulting in hypercontractility, diastolic dysfunction, and impaired energetics (B). CMIs such as mavacamten and aficamten stabilize the SRX state, reduce excessive cross-bridge cycling, and restore energetic efficiency (C). Schematic chemical structures of mavacamten and aficamten are shown (D). Created in Illustrae (https://illustrae.co/)

Selective CMIs, including mavacamten and aficamten, act by stabilizing the SRX state and reducing the number of myosin heads available for cross-bridge cycling (Fig. 2). This allosteric modulation of myosin ATPase activity normalizes contractility, improves diastolic filling, and reduces LVOT gradients [6]. Unlike traditional negative inotropes, which blunt adrenergic signaling (β-blockers), calcium entry (verapamil), or sodium conductance (disopyramide), CMIs directly target the sarcomere, providing the first disease-specific pharmacological therapy for HCM. Disease-modifying preclinical studies demonstrated that chronic myosin inhibition not only corrects hypercontractility but also attenuates pathological hypertrophy and fibrosis, restoring myocardial energetics and efficiency [16]. Importantly, early human data from cardiac magnetic resonance imaging (MRI) substudies suggest reductions in left ventricular mass and improvements in filling dynamics during long-term therapy [17]. These findings raise the possibility that CMIs may act not only as symptom-modifying but also as disease-modifying therapies, capable of altering the natural trajectory of HCM.

## 3. Clinical Development of Myosin Inhibitors 

The translation of myosin inhibition from mechanistic insight to clinical application has been remarkable. Within little more than a decade, selective CMIs have advanced from preclinical proof-of-concept to phase 3 trials demonstrating meaningful clinical benefit in obstructive HCM.

### 3.1 Mavacamten 

The first-in-class inhibitor, mavacamten, was initially evaluated in the PIONEER-HCM trial, an open-label phase 2 study in symptomatic obstructive HCM patients [18, 19]. PIONEER demonstrated for the first time that pharmacological sarcomere modulation could achieve significant reductions in LVOT gradients, improve exercise capacity, and favourably remodel cardiac structure [18]. These results established the feasibility of myosin inhibition as a disease-specific therapeutic strategy. The subsequent EXPLORER-HCM trial, a global, randomized, double-blind, placebo-controlled phase 3 study, confirmed and extended these findings [20]. In EXPLORER, the largest randomized trial on HCM when it was conducted, mavacamten achieved the primary endpoint of improving exercise capacity and symptoms, with 37% of patients reaching the composite endpoint versus 17% in the placebo group. Moreover, nearly three-quarters of patients experienced improvement of at least one New York Heart Association (NYHA) functional class, paralleled by significant reductions in LVOT gradient, N-terminal pro-B-type natriuretic peptide (NT-proBNP), and troponin levels. Importantly, quality of life scores (Kansas City Cardiomyopathy Questionnaire, KCCQ) improved substantially, underscoring the clinical relevance of symptom relief [20]. Beyond symptomatic benefit, the VALOR-HCM trial provided evidence that myosin inhibition may alter established treatment pathways in obstructive HCM. In this randomized phase 3 study of patients referred for septal reduction therapy (SRT), only 18% of those receiving mavacamten remained eligible for SRT at week 16, compared with 77% in the placebo group [21, 22]. This dramatic reduction highlights the capacity of mavacamten to defer or even obviate the need for invasive septal reduction procedures, which have long been the cornerstone of management in advanced obstructive HCM. Importantly, these clinical improvements were accompanied by consistent reductions in resting and Valsalva LVOT gradients, NT-proBNP levels, and troponin concentrations, underscoring both hemodynamic and biomarker evidence of therapeutic benefit [21, 22].

### 3.2 Aficamten 

While mavacamten validated the therapeutic concept, its pharmacokinetic profile necessitates regular echocardiographic monitoring due to its long half-life time, a relatively narrow therapeutic window and potential for relative overdosing with consecutive left ventricular systolic dysfunction. Aficamten, a next-generation CMI, was designed to overcome these limitations (Table 1). In the REDWOOD-HCM phase II trial, aficamten demonstrated dose-dependent, rapid, and reversible reductions in LVOT gradients, paralleled by improvements in symptoms and biomarkers, with an excellent safety profile [23, 24]. The SEQUOIA-HCM phase III trial has also established aficamten as an effective therapy in symptomatic obstructive HCM [25]. At 24 weeks, aficamten significantly improved exercise capacity (between-group difference in peak VO_2_: +1.7 mL/kg/min), health status (Kansas City Cardiomyopathy Questionnaire-Clinical Summary Score (KCCQ-CSS): +7 points vs placebo), and NYHA class (58.5% vs 24.3% improved), while achieving near-complete gradient relief in almost half of treated patients [25]. The MAPLE-HCM trial provided the first direct, head-to-head comparison of a CMI with standard-of-care beta-blockade [26, 27]. In this phase III, double-blind study, 175 untreated patients with symptomatic obstructive HCM were randomized to aficamten (5–20 mg quaque die once daily, titrated) or metoprolol (dosed to 50–200 mg QD) for 24 weeks [27]. Aficamten was superior to metoprolol for the primary endpoint of peak VO_2_, and demonstrated consistent benefits across key secondary endpoints: NYHA class improvement, KCCQ-CSS (+6.9 points), Valsalva LVOT gradient (–34.9 mmHg), NT-proBNP (ratio 0.19), and left atrial volume index (-7 mL/m^2^). Importantly, after a 4-week washout, physiologic effects waned, consistent with aficamten’s short half-life and pharmacodynamic reversibility. The safety profile was acceptable, with left ventricular ejection Fraction (LVEF) <50% in <5% of patients, all reversible [27]. Taken together, MAPLE-HCM demonstrates that aficamten is not only effective in refractory or add-on settings but can outperform standard first-line therapy, positioning CMIs as potential frontline treatment for obstructive HCM. This represents a critical step in moving sarcomere-directed therapy from a “specialized option” toward changing the default standard of care in obstructive HCM. However, it remains uncertain whether this pharmacokinetic profile confers any long-term clinical advantages over mavacamten, and this requires further investigation.

**Table 1.** **Comparison of cardiac myosin inhibitors: mavacamten vs aficamten**.

Characteristic	Mavacamten (Camzyos®)	Aficamten
Molecular class	Small-molecule, allosteric cardiac myosin inhibitor (1st generation)	Small-molecule, allosteric cardiac myosin inhibitor (2nd generation)
Binding/Mechanism	Stabilizes super-relaxed state; reduces cross-bridge cycling	Same mechanism, but distinct allosteric binding site; designed for wider therapeutic window
Half-life (t½)	∼6–9 days in normal CYP2C19 metabolizers; up to ∼23 days in poor metabolizers	∼3.4–3.5 days
Time to steady state	∼6 weeks	∼2 weeks
Wash-out	Requires ∼5 half-lives: ∼45 days (normal metabolizers), up to 115 days in poor metabolizers	Faster wash-out due to shorter t½; reversibility within days–weeks
Metabolism	Primarily CYP2C19, minor CYP3A4	Minimal CYP involvement
Drug–drug interactions	Contraindicated with strong CYP2C19/CYP3A4 inhibitors or inducers	Low DDI risk reported in trials
Dose titration	Tablets 2.5/5/10/15 mg once daily; titration based on LVOT gradient & LVEF	5–20 mg once daily in trials; echo-guided titration
Monitoring requirements	Mandatory REMS program (serial echocardiography)	May require less intensive long-term monitoring, no REMS (not yet approved)
Key efficacy (Phase III)	EXPLORER-HCM, VALOR-HCM: Significant improvements in exercise capacity, symptoms, and quality of life; marked LVOT gradient reduction; reduced eligibility for septal reduction therapy	SEQUOIA-HCM: Significant improvements in exercise capacity, symptoms, and quality of life; marked LVOT gradient reduction, comparable to mavacamten
Safety (LVEF <50%)	6–14% across trials; all reversible	∼4–7% across trials; all reversible
Regulatory status (2025)	FDA (2022) and EMA (2023) approved for symptomatic oHCM	NDA under FDA review (PDUFA Dec 2025)

CYP, Cytochrome P450; DDI, drug–drug interaction; EMA, European Medicines Agency; FDA, U.S. Food and Drug Administration; LVEF, left ventricular ejection fraction; LVOT, left ventricular outflow tract; NDA, New Drug Application; oHCM, obstructive hypertrophic cardiomyopathy; PDUFA, Prescription Drug User Fee Act date; REMS, Risk Evaluation and Mitigation Strategy.

### 3.3 Non-Obstructive HCM 

While most drug development has centered on obstructive HCM, nearly one-third of patients present with non-obstructive HCM. These patients experience substantial symptom burden and progressive functional limitation, yet no disease-specific therapies are currently approved. Conventional agents such as β-blockers, calcium channel blockers, or diuretics provide only partial relief, and treatment remains largely supportive. Trials of phase 2 for valsartan-sacubitril also could not show clinical benefit [28]. The first dedicated exploration of myosin inhibition in this population was the MAVERICK-HCM trial, which demonstrated that mavacamten therapy induced significant reductions in biomarkers of wall stress (NT-proBNP) and myocardial injury (high-sensitivity troponin), establishing biological proof-of-concept even in the absence of predefined clinical endpoints [29]. More recently, REDWOOD-HCM Cohort 4 extended this evidence to aficamten, reporting clinically meaningful improvements in symptoms, quality of life (KCCQ scores), and reductions in NT-proBNP and troponin [23]. ODYSSEY-HCM was a randomized, double-blind, placebo-controlled phase III trial evaluating mavacamten in patients with symptomatic non-obstructive HCM. This trial (n = 580) did not meet its co-primary endpoints at 48 weeks: the between-group differences in peak VO_2_ (Δ 0.47 mL/kg/min; *p* = 0.07) and KCCQ-CSS (Δ 2.7 points; *p* = 0.06) were borderline statistically significant, despite marked reductions in NT-proBNP and troponin with mavacamten. This apparent dissociation between biomarker improvement and functional outcomes likely reflects the pathophysiology of non-obstructive HCM, which may require longer-term structural remodeling before producing measurable gains in peak VO₂ or quality of life. Additionally, protocol-guided treatment interruptions were more frequent with mavacamten (26% vs 8%), and LVEF <50% occurred more often than with placebo (22% vs 2%), with recovery after interruption in almost all cases [30]. This higher incidence likely reflects the ODYSSEY titration strategy, in which dosing was escalated to the highest tolerated level in the absence of a gradient-based target, unlike obstructive HCM trials where LVOT-guided titration limits excessive negative inotropic exposure. Together, these results temper clinical expectations for CMIs in non-obstructive HCM, at least in the investigated short-term setting that does not cover the effects related to longer-term remodelling. It also underscores the need for careful phenotype-specific patient selection and monitoring. The ongoing ACACIA-HCM program will clarify whether aficamten confers clinically meaningful benefit in this population.

## 4. Practical Considerations

The introduction of CMIs into clinical practice has been accompanied by close attention to safety, tolerability, and monitoring requirements. While both mavacamten and aficamten demonstrate consistent efficacy across pivotal trials, their pharmacokinetic properties, potential for left ventricle (LV) systolic dysfunction, and regulatory frameworks necessitate careful implementation. A key safety consideration is the potential for transient reductions in LVEF, reflecting the intended negative inotropic mechanism. In EXPLORER-HCM, VALOR-HCM, and MAVA-LTE, between 5% and 14% of mavacamten-treated patients experienced a reduction in LVEF <50% [20, 22, 31]. In all cases managed per protocol with drug interruption or dose adjustment, systolic function recovered, underscoring the reversibility of this effect. Importantly, all cases managed per protocol with temporary drug interruption or dose adjustment recovered, underscoring the predictable and reversible nature of this effect. By contrast, aficamten’s shorter terminal half-life (about 3.5 days) facilitates more rapid pharmacodynamic reversibility. In REDWOOD-HCM, SEQUOIA-HCM, and FOREST-HCM, transient reductions in LVEF <50% occurred in approximately 3–7% of patients, were consistently reversible with dose adjustment or interruption, and were not associated with clinical heart failure [24, 25, 32]. Notably, in the non-obstructive HCM population enrolled in ODYSSEY-HCM, LVEF <50% occurred more frequently with mavacamten than with placebo (22% vs 2%), highlighting phenotype-specific safety considerations in non-obstructive disease [30]. These findings support the need for structured echocardiographic monitoring during dose titration for both mavacamten and aficamten, as patients receiving either agent remain at risk for transient reductions in LVEF. Although aficamten’s shorter half-life may allow faster pharmacodynamic reversibility, this does not eliminate the need for regular monitoring. However, modest reductions in LVEF observed with both mavacamten and aficamten are an expected pharmacodynamic effect of myosin inhibition and reflect target engagement rather than myocardial injury. These decreases are typically mild, reversible with dose adjustment or brief interruption, and rarely associated with symptoms. A lower incidence of LVEF reduction should not be interpreted as evidence of greater long-term disease-modifying potency. Furthermore, disease stage may influence susceptibility to systolic dysfunction during CMI therapy. Patients with advanced HCM phenotypes, such as those with extensive myocardial fibrosis or borderline systolic function, may theoretically be more vulnerable to LVEF reduction. Further subgroup analyses and dedicated prospective studies are needed to determine whether these factors modify the susceptibility to LVEF reduction during CMI therapy.

Mavacamten is metabolized primarily via Cytochrome P450 2C19 (CYP2C19) and CYP3A4, creating potential for drug-drug interactions with agents such as antiarrhythmics, antifungals, and proton-pump inhibitors [33]. Accordingly, comprehensive medication reconciliation is essential prior to and during therapy. In a recent analysis of real-world data of a post-market approval registry in the US, 99% of patients did not show any clinically relevant interacting medication before initiation of mavacamten. Aficamten, while not yet approved, undergoes minimal CYP-mediated metabolism, which may improve safety in elderly patients and those with polypharmacy [33].

Overall, CMIs have shown a favorable tolerability profile. The most frequently reported adverse events include dizziness, fatigue, and transient reductions in ejection fraction, with no evidence of excess arrhythmias or proarrhythmic risk compared with placebo. Importantly, treatment discontinuation due to adverse events remains low (<5%) [31, 32, 34]. Pregnancy and breastfeeding remain areas of caution, as safety data are insufficient; current recommendations advise avoiding CMIs in women of childbearing potential unless effective contraception is ensured [35].

## 5. Challenges and Controversies

Despite their promise, several important uncertainties and challenges remain in the clinical implementation of CMIs. While MAVA-LTE and FOREST-HCM provide reassuring data over 2–3 years, the long-term consequences of chronic sarcomere inhibition are unknown. Whether lifelong therapy in young patients alters survival, sudden cardiac death risk, or progression to end-stage heart failure remains unanswered. In patients otherwise eligible for SRT, whether CMIs should be offered as first-line alternatives or as a bridge remains debated. VALOR-HCM demonstrated a striking reduction in SRT eligibility, but head-to-head comparisons with surgical myectomy or alcohol septal ablation are lacking [22]. CMI use after acute myocardial infarction raises concern: transient negative inotropy in a myocardium already compromised by ischemic injury could exacerbate pump failure. No study has systematically evaluated post-myocardial infarction (MI) use, and this remains a relative contraindication until further evidence emerges. Similarly, in overdose scenarios, the predictable risk is profound systolic dysfunction; while reversible, optimal management strategies (temporary mechanical support, pharmacological reversal) remain theoretical and require guideline development. Not all HCM patients harbor sarcomere mutations, and treatment response may differ by genotype. Preliminary analyses suggest that sarcomere-positive patients may derive greater structural reverse remodeling, but robust genotype–phenotype–treatment interaction data are limited. Precision cardiology approaches incorporating genetics, imaging, and biomarkers will be essential to refine patient selection. CMIs can be used safely in patients with mild-to-moderate renal impairment (estimated Glomerular Filtration Rate, eGFR ≥30), but their safety and efficacy in dialysis-dependent end-stage kidney disease are unstudied and application cannot currently be recommended in dialysis-dependent patients [33]. Dedicated pharmacokinetic and clinical outcome trials are urgently needed to inform dosing, safety, and monitoring strategies in this vulnerable population. High drug costs represent another barrier, particularly in healthcare systems where invasive septal reduction is available and reimbursed. Economic analyses are needed to ensure sustainable, equitable global access. While both mavacamten and aficamten have shown robust efficacy and favorable safety profiles, definitive conclusions about comparative clinical advantages cannot be drawn without a dedicated head-to-head randomized trial. A study designed with safety as the primary endpoint, now that efficacy has been established, would be valuable to determine whether pharmacokinetic differences, such as the shorter half-life of aficamten, translate into meaningful advantages in real-world patient management.

## 6. Future Directions and Conclusion

The therapeutic horizon for cardiac myosin inhibition is rapidly expanding. Ongoing studies will determine whether the symptomatic and biomarker improvements observed in early-phase studies translate into robust functional and quality-of-life benefits in non-obstructive HCM. Parallel pediatric and adolescent studies (e.g., CEDAR, SCOUT) are evaluating safety and efficacy in younger patients, where early intervention may prevent maladaptive remodeling and alter lifetime disease trajectory. Beyond classical HCM, CMIs may also have a role in other conditions of hypercontractility. Early exploratory work suggests potential application in heart failure with preserved ejection fraction (HFpEF), where sarcomeric hypercontractility and diastolic dysfunction contribute to pathophysiology [36]. Further investigation will determine whether CMIs can extend benefit into this broader heart failure population.

Integration of CMIs with genetic testing and precision cardiology frameworks could ultimately enable targeted therapy for at-risk mutation carriers before overt disease manifests, opening the door to disease prevention. In parallel, real-world registries and long-term extension studies will be essential to address questions of durability, late safety signals, and cost-effectiveness, and to define their impact on hard outcomes such as progression to heart failure, arrhythmia burden, and mortality.

The positioning of CMIs within current treatment algorithms is becoming increasingly relevant. β-blockers and non-dihydropyridine calcium channel blockers remain the recommended first-line therapy for symptomatic obstructive HCM, with CMIs and, if symptoms persist, disopyramide or SRT considered as subsequent treatment options. As evidence continues to mature, CMIs may increasingly be used as an early-line option in obstructive HCM. In non-obstructive HCM, their role remains investigational, and careful phenotype-specific patient selection is essential. Integration of CMIs into clinical practice should rely on shared decision-making, taking into account patient goals, comorbidities, and treatment availability.

In conclusion, cardiac myosin inhibitors represent the first truly disease-specific pharmacological therapy for hypertrophic cardiomyopathy. By directly modulating sarcomeric hypercontractility, CMIs achieve outcomes previously attainable only through invasive septal reduction procedures: relief of obstruction, symptomatic improvement, and evidence of structural reverse remodeling. As development extends to non-obstructive disease, pediatric populations, and next-generation molecules, CMIs are poised to fundamentally redefine the standard of care in HCM. While challenges remain—including uncertainties about long-term outcomes, equitable access, and patient selection—myosin inhibition stands as a revolutionary game changer, bridging decades of genetic discovery with tangible clinical translation.
